# Coexistence of Communicating and Noncommunicating Cells in the Filamentous Cyanobacterium *Anabaena*

**DOI:** 10.1128/mSphere.01091-20

**Published:** 2021-01-13

**Authors:** Sergio Arévalo, Anja Nenninger, Mercedes Nieves-Morión, Antonia Herrero, Conrad W. Mullineaux, Enrique Flores

**Affiliations:** a Instituto de Bioquímica Vegetal y Fotosíntesis, Consejo Superior de Investigaciones Científicas and Universidad de Sevilla, Seville, Spain; b School of Biological and Chemical Sciences, Queen Mary University of London, London, United Kingdom; University of Iowa

**Keywords:** cyanobacteria, intercellular communication, septal junctions

## Abstract

In filamentous heterocyst-forming (N_2_-fixing) cyanobacteria, septal junctions join adjacent cells, mediating intercellular communication, and are thought to traverse the septal peptidoglycan through nanopores. Fluorescence recovery after photobleaching (FRAP) analysis with the fluorescent marker calcein showed that cultures of *Anabaena* sp. strain PCC 7120 grown in the presence of combined nitrogen contained a substantial fraction of noncommunicating cells (58% and 80% of the tested vegetative cells in nitrate- and ammonium-grown cultures, respectively), whereas cultures induced for nitrogen fixation contained far fewer noncommunicating cells (16%). A single filament could have communicating and noncommunicating cells. These observations indicate that all (or most of) the septal junctions in a cell can be coordinately regulated and are coherent with the need for intercellular communication, especially under diazotrophic conditions. Consistently, intercellular exchange was observed to increase in response to N deprivation and to decrease rapidly in response to the presence of ammonium in the medium or to nitrate assimilation. Proteins involved in the formation of septal junctions have been identified in *Anabaena* and include SepJ, FraC, and FraD. Here, we reevaluated rates of intercellular transfer of calcein and the number of nanopores in mutants lacking these proteins and found a strong positive correlation between the two parameters only in cultures induced for nitrogen fixation. Thus, whereas the presence of a substantial number of noncommunicating cells appears to impair the correlation, data obtained in diazotrophic cultures support the idea that the nanopores are the structures that hold the septal junctions.

**IMPORTANCE** Multicellularity is found in bacteria as well as in eukaryotes, and the filamentous heterocyst-forming (N_2_-fixing) cyanobacteria represent a simple and ancient paradigm of multicellular organisms. Multicellularity generally involves cell-cell adhesion and communication. The cells in the cyanobacterial filaments are joined by proteinaceous septal junctions that mediate molecular diffusion. The septal junctions traverse the septal peptidoglycan, which bears holes termed nanopores. Our results show that the septal junctions can be coordinately regulated in a cell and emphasize the relationship between septal junctions and nanopores to build intercellular communication structures, which are essential for the multicellular behavior of heterocyst-forming cyanobacteria.

## Abstract (precis)

Multicellularity is found in bacteria as well as in eukaryotes, and the filamentous heterocyst-forming (N_2_-fixing) cyanobacteria represent a simple and ancient paradigm of multicellular organisms. Multicellularity generally involves cell-cell adhesion and communication.

## INTRODUCTION

Multicellularity is a way of organizing living matter, in which an organism is composed of multiple cells that communicate through intercellular molecular exchange. Because multicellularity generally involves the differentiation of cells to perform specialized tasks, intercellular communication plays a key role for the behavior of the body of cells as an organismic unit (1). Multicellularity is evidently found in eukaryotes but also in prokaryotes, and outstanding examples of the latter are the filamentous heterocyst-forming cyanobacteria. These organisms grow as filaments that can be made of hundreds of cells and in which, under nitrogen deprivation, two cell types are found: the vegetative cells that fix CO_2_, performing oxygenic photosynthesis, and the heterocysts that are specialized for the fixation of atmospheric N_2_. Heterocysts represent approximately 10% of the cells in a filament and provide the vegetative cells with fixed nitrogen (2), which is transferred in the form of amino acids and a dipeptide (3, 4). In turn, because the heterocysts do not fix CO_2_ photosynthetically, they are provided by the vegetative cells with fixed carbon (5), mainly in the form of a sugar such as sucrose (6–8). Because one heterocyst relates to several vegetative cells, this exchange of nutrients requires molecular exchange not only between heterocysts and vegetative cells but also between vegetative cells in a row. Additionally, inhibitors of heterocyst differentiation, which are required for establishing the heterocyst distribution pattern along the filament, are transferred from heterocysts or prospective heterocysts to vegetative cells, reaching several cell units away from the inhibitor source (9, 10).

Intercellular molecular exchange in filamentous cyanobacteria can be studied by fluorescence recovery after photobleaching (FRAP) using fluorescent markers such as calcein (11). Intercellular molecular exchange takes place by diffusion, presumably through conduits that connect the cytoplasm of adjacent cells (11, 12). In isolated peptidoglycan (PG; murein) sacculi of heterocyst-forming cyanobacteria, septal peptidoglycan disks can be identified that present holes that have been termed nanopores (13). Three key cytoplasmic membrane (CM) proteins of polar localization, SepJ, FraC, and FraD, which are necessary to make long filaments, have been identified in the model heterocyst-forming cyanobacterium *Anabaena* sp. strain PCC 7120 (1, 14). Mutants lacking these proteins are impaired in intercellular molecular exchange and in the formation of nanopores (8, 11, 15). Hence, they are thought to be involved in the production of proteinaceous structures, termed septal junctions (formerly “microplasmodesmata”), that mediate intercellular diffusion (1). Whereas *sepJ* inactivation mutants show the strongest known filament fragmentation phenotype, implying that SepJ is essential to form mature septa, FraC and FraD are related to septal junctions that have been visualized by cryoelectron tomography in the filaments of *Anabaena*, in which FraD is a structural component and FraC is involved in their assembly (16). The septal junctions are composed of a tube connecting the adjacent cells and a plug and a cap on the cytoplasmic membrane. In response to stress, a structural rearrangement of the septal junction caps has been observed that results in loss of communication between cells and is reversible upon stress avoidance, suggesting a mechanism of gating for these septal junctions (16).

To attain a better understanding of the relation between nanopores and intercellular molecular exchange mediated by the septal junctions, here, we reevaluated nanopore numbers and rates of intercellular transfer of calcein in *Anabaena* and mutants lacking the septal proteins SepJ, FraC, and FraD. Cells that showed little or no communication activity were found to coexist with cells showing substantial communication, and their proportion depended on the nitrogen nutrition of the filaments. Furthermore, we show that intercellular molecular exchange responds rapidly to the supply of combined nitrogen nutrients.

## RESULTS

### Nanopores.

**FIG 1:**
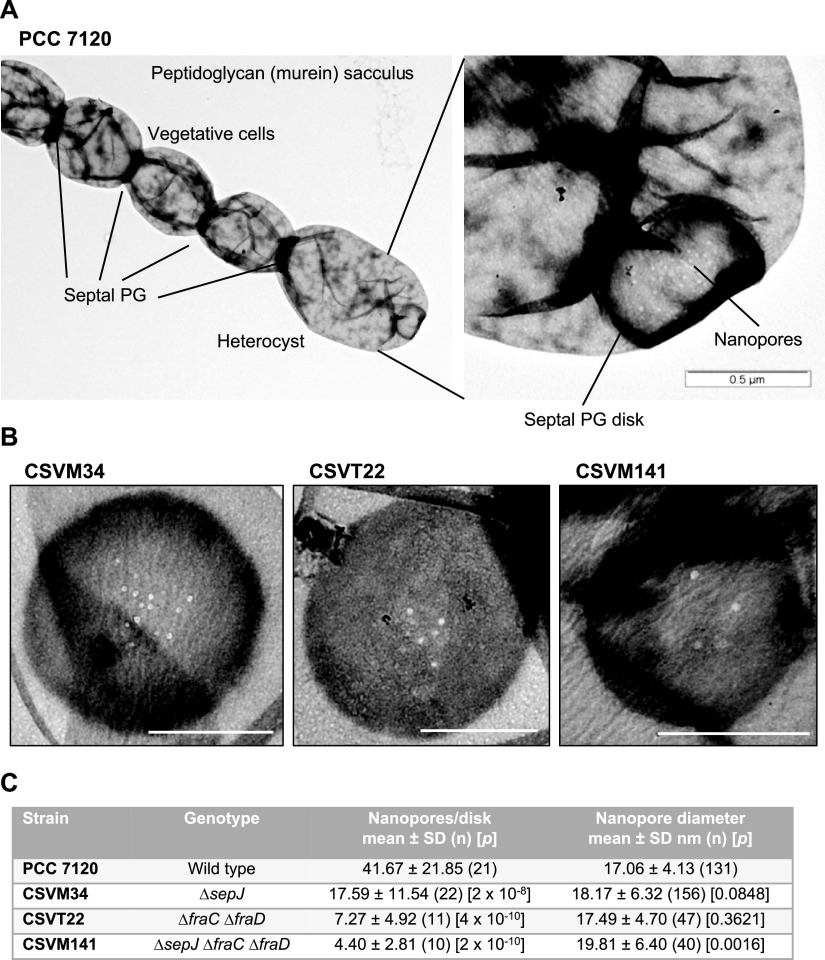
Nanopores in septal peptidoglycan disks of *Anabaena*. (A) Peptidoglycan (murein) sacculus of part of a filament of *Anabaena* grown in BG11_0_ medium. The PG was isolated and visualized by transmission electron microscopy as described in Materials and Methods. Note the thickness of septal PG, the presence of one heterocyst, and a septal PG disk with nanopores. (B) Nanopores in disks from *Anabaena* mutants CSVM34, CSVT22, and CSVM141 grown in BG11 medium. Scale bars, 500 nm. (C) Genotypes of the strains analyzed and means and standard deviations of nanopore numbers and diameters. *n*, number of septal disks (nanopore counting) or number of nanopores (diameter) analyzed. The difference between each mutant and the wild type was assessed by the Student’s *t* test (*P* values are indicated).

PG sacculi of *Anabaena* strains grown in BG11 medium (containing nitrate as the nitrogen source) or BG11_0_ medium (without combined nitrogen, which elicits heterocyst differentiation) were isolated, stained with uranyl acetate, and visualized by transmission electron microscopy as previously described (8, 13, 17) (see also Materials and Methods). Figure 1A shows a murein sacculus corresponding to 5 cells of a filament of *Anabaena* including a heterocyst, identified by its larger size. (To the best of our knowledge, this is the first time that a multicellular sacculus including a heterocyst has been shown.) Especially electrodense PG can be appreciated at the septa, likely reflecting the juxtaposition of the PG layers of the adjacent cells. The fact that those cells remain bound after isolation implies that the PG layers of adjacent cells are chemically linked. In the exposed heterocyst pole, a septal PG disk showing nanopores is clearly visualized.

The number of nanopores and their diameters were reevaluated in *Anabaena* mutants Δ*sepJ* (strain CSVM34 [18]), Δ*fraC* Δ*fraD* (CSVT22 [19]), and Δ*sepJ* Δ*fraC* Δ*fraD* (CSVM141 [8]). Figure 1B shows representative examples of septal PG disks from these mutants. The frequency distributions of nanopores show decreased nanopore numbers in strains CSVM34, CSVT22, and CSVM141 (see Fig. S1A in the supplemental material). Nanopore numbers, summarized in Fig. 1C, are similar to those described by Nürnberg et al. (8), except for strain CSVM34, which had approximately 42% of the nanopore number in the wild type, in contrast to 24% described previously (8). The frequency distributions of nanopore diameters were broadly similar for the wild type and the three mutants (Fig. S1B), but quantitative analysis showed that nanopore diameters were significantly larger in the triple mutant (strain CSVM141) than in the wild type (Student’s *t* test, *P* < 0.01) (Fig. 1C). In contrast to the study described previously (8), in the present analysis, we did not find a significant difference in nanopore diameters between the Δ*sepJ* (strain CSVM34) or Δ*fraC* Δ*fraD* (strain CSVT22) mutant and the wild type (Student’s *t* test, *P* > 0.05) (Fig. 1C).

### Intercellular calcein transfer.

For FRAP analysis, cells are loaded with a fluorescent marker, one cell in a filament is bleached, and the recovery of fluorescence in the bleached cell (due to marker diffusion from neighboring cells) and loss of fluorescence in the neighboring cells are followed by confocal microscopy, allowing the determination of exchange coefficients (*E*) or recovery rate constants (*R*) (11, 19). These parameters have generally been described as the means and standard deviations of sets of data, which assumes a normal distribution of the data. However, it has been recently noted that some mutants of *Anabaena* present a substantial number of cells that do not recover from bleaching; such cells show *R* values of less than 0.01 s^−1^ and have been defined as noncommunicating cells (14). (Indeed, most of those cells showed *R* values of <0.001 s^−1^, but we chose to define noncommunicating cells as those with an *R of* ≤0.01 s^−1^ to facilitate representation and analysis of the data [14].) Here, we analyzed intercellular molecular exchange by studying calcein FRAP performed as previously described (11), and the results are presented as the recovery rate constant (*R*) that was calculated as previously reported (20). Filaments of *Anabaena* were grown under standard photoautotrophic conditions in BG11 medium (or, when indicated, BG11_0_ plus NH_4_^+^ medium), or were grown in BG11 medium and incubated for 48 h in BG11_0_ medium (21) (see also Materials and Methods).

**FIG 2:**
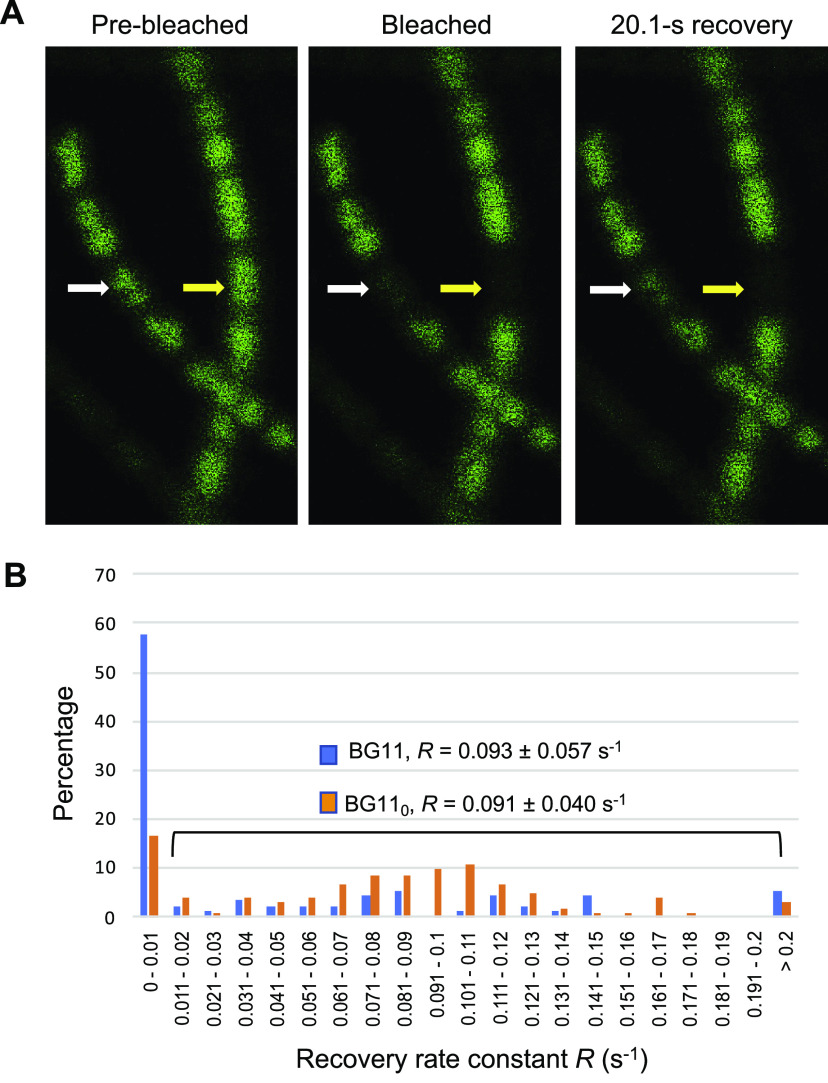
Calcein FRAP analysis. (A) Example of FRAP analysis of two filaments of the same culture (BG11 medium). The bleached cell showed recovery of fluorescence in one of the filaments (white arrow) but not in the other (yellow arrow). (B) Intercellular transfer of calcein between vegetative cells of *Anabaena* sp. PCC 7120 grown in BG11 medium (blue) or incubated for 48 h in BG11_0_ medium (orange). Filaments were labeled with calcein and subjected to FRAP analysis as described in Materials and Methods. Data are organized in groups according to *R* values in 0.01-s^−1^ increments. Five cells from BG11 medium gave *R* values of >0.2 s^−1^ (0.247, 0.266, 0.312, 0.436, and 0.505 s^−1^), and three cells from BG11_0_ medium gave *R* values of >0.2 s^−1^ (0.227, 0.327, and 0.861 s^−1^). *R* values of >0.3 s^−1^ were not included in further analysis, because they may correspond to not fully divided cells (see the main text). A total of 90 cells from 7 independent cultures in BG11 medium and a total of 103 cells from 8 independent cultures in BG11_0_ medium were analyzed. Some of these cultures correspond to the wild-type controls used in previously published studies (21, 23). Noncommunicating cells were found in all of the 7 BG11 cultures and in 5 of the 8 BG11_0_ cultures.

We found that most cultures contained cells that, after bleaching in the FRAP analysis, recovered fluorescence as expected, but we also found cells that did not recover fluorescence (Fig. 2A). BG11-grown cultures of the wild type had a substantial number (approximately 58%) of noncommunicating cells (*R* ≤ 0.01 s^−1^) (Fig. 2B). On the other hand, a few cells showed outrageously high *R* values, e.g., cells with *R* values of >0.3 s^−1^ (see the legend to Fig. 2). Because such high *R* values are similar to recovery times expected for free diffusion in the cytoplasm (22), in these cells, the cytoplasm might be not fully divided from that of a daughter cell (and perhaps further neighboring cells) after cell division; thus, these cells were not included in further analysis. In contrast to cells grown in BG11 medium, the cultures incubated in BG11_0_ medium had fewer noncommunicating cells (approximately 16%). The increase in the fraction of communicating cells in the cultures incubated in BG11_0_ medium can represent a significant aspect of the acclimation of *Anabaena* to diazotrophic growth. As a further test, BG11_0_ plus NH_4_^+^-grown filaments were investigated and found to contain 80% noncommunicating cells (*n* = 66, from two independent cultures).

When the *R* values were calculated omitting noncommunicating cells (*R* ≤ 0.01 s^−1^), very similar recovery constants were obtained for cells grown in BG11 medium and those incubated in BG11_0_ medium (Fig. 2B). This indicates that communicating cells have similar communication properties in both types of cultures. These recovery constants were also very similar to those previously reported for BG11-grown wild-type cells (8), suggesting that the cultures analyzed previously either did not contain noncommunicating cells or such cells were somehow missed. It is possible that, in addition to the nitrogen source, the fraction of noncommunicating cells depends on other growth parameters (temperature, light quality and intensity, and CO_2_ supply), which may explain why they were not noticed previously.

To test whether a single filament of *Anabaena* could have both communicating and noncommunicating cells, we performed calcein FRAP analysis in two cells (and in a few cases, three cells) separated by intervals of 6 to 9 cells in the same BG11-grown filament. A total of 44 cells from 20 different filaments were subjected to FRAP analysis, and we found that in 10 filaments (50%), one of the tested cells was communicating and the other tested cell was noncommunicating. Additionally, none of the tested cells were communicating in seven filaments (35%), and the two tested cells were communicating in three filaments (15%). In similar experiments performed with BG11_0_ plus NH_4_^+^-grown cells, in which most cells were noncommunicating, 5 of 22 tested filaments (23%) contained at least one communicating cell in addition to a noncommunicating cell(s). Thus, our results show that a single filament can have both communicating and noncommunicating cells.

### Intercellular transfer in septal protein mutants.

**FIG 3:**
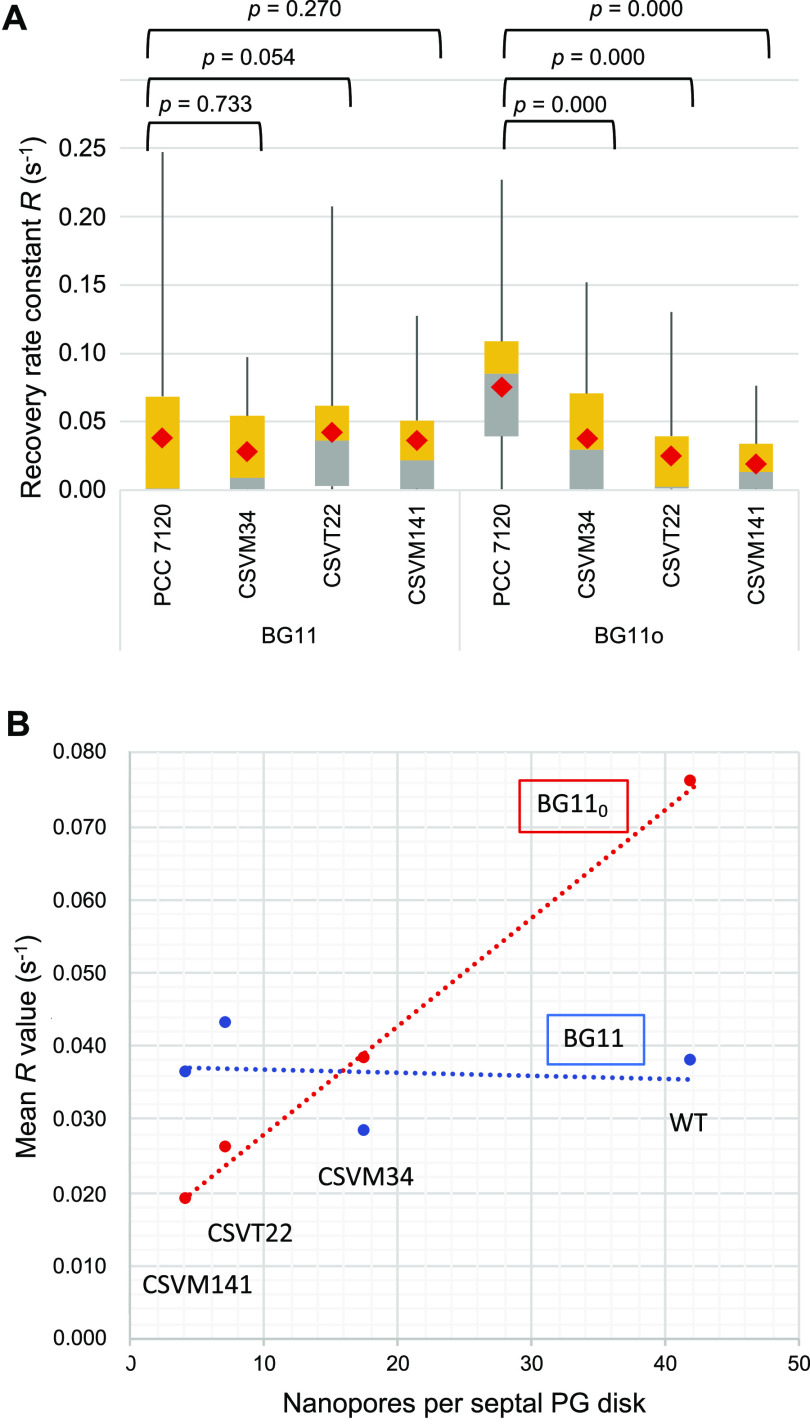
Intercellular molecular transfer and its correlation with nanopore numbers in *Anabaena* and some septal protein mutants. (A) Box plot representation and statistical analysis of calcein transfer between vegetative cells of *Anabaena* and mutant strains CSVT22 (Δ*fraC* Δ*fraD*), CSVM34 (Δ*sepJ*), and CSVM141 (Δ*fraC* Δ*fraD* Δ*sepJ*) grown in BG11 medium or incubated for 48 h in BG11_0_ medium. The difference between each mutant and the wild type was assessed by the Mann-Whitney U test (*P* values shown at the top). Gray, quartile group 2 (from Q1 to median); yellow, quartile group 3 (from median to Q3); red diamonds, mean values. (B) Nanopore numbers and activity of calcein transfer between vegetative cells of *Anabaena* wild type (WT) and mutant strains CSVM34, CSVT22, and CSVM141 grown in BG11 medium or incubated for 48 h in BG11_0_ medium.

We then addressed calcein FRAP in the deletion mutants of the septal protein-encoding genes *sepJ*, *fraC*, and *fraD*. Significant numbers of noncommunicating cells were observed for all the mutants not only in BG11-grown filaments but also in filaments that had been incubated in BG11_0_ medium (see Fig. S2). To compare the mutants to the wild type, we represented calcein transfer rates in box plots and, given the nonparametric distribution of the data, used the Mann-Whitney U test to assess the significance of the difference between each mutant and the wild type (Fig. 3A). This analysis, which also takes into account noncommunicating cells (*R* ≤ 0.01 s^−1^), shows that in BG11 medium, there were no significant differences between any of the mutants and the wild type, whereas in BG11_0_ medium, the three mutants were significantly different from the wild type, showing sets of *R* values lower than those of the wild type. These results differ from those previously described for the mutants grown in BG11 medium but confirm the results obtained with filaments that had been incubated in the absence of combined nitrogen (11, 15). The difference with previously described analyses may rely on the high number of noncommunicating cells that we have found in the wild type grown with combined nitrogen.

### Nanopores and intercellular calcein transfer.

Similar numbers of nanopores are found in *Anabaena* grown in BG11 and in BG11_0_ medium (17, 23). Here, we compared nanopore numbers and the rates of intercellular calcein transfer in *Anabaena* (wild type) and in the *sepJ*, *fraC*, and *fraD* deletion mutants. (For calcein transfer, data with an *R* of >0.3 s^−1^ were excluded from analysis as explained above.) Intercellular calcein transfer rate and nanopore number showed a strong correlation in filaments that had been incubated without combined nitrogen (BG11_0_ medium, correlation coefficient = 0.9984) but not in filaments grown with combined nitrogen (BG11 medium, correlation coefficient = −0.1083) (Fig. 3B). The low rate of calcein transfer in the mutants appears to result mainly from a shortage of nanopores (see Discussion). In contrast, in the wild type, the decreased rate of calcein transfer in BG11 medium compared to that in BG11_0_ medium appears to result from the presence of cells that, despite containing a similar number of nanopores, were noncommunicating.

### Response to changes in nitrogen nutrition.

**FIG 4:**
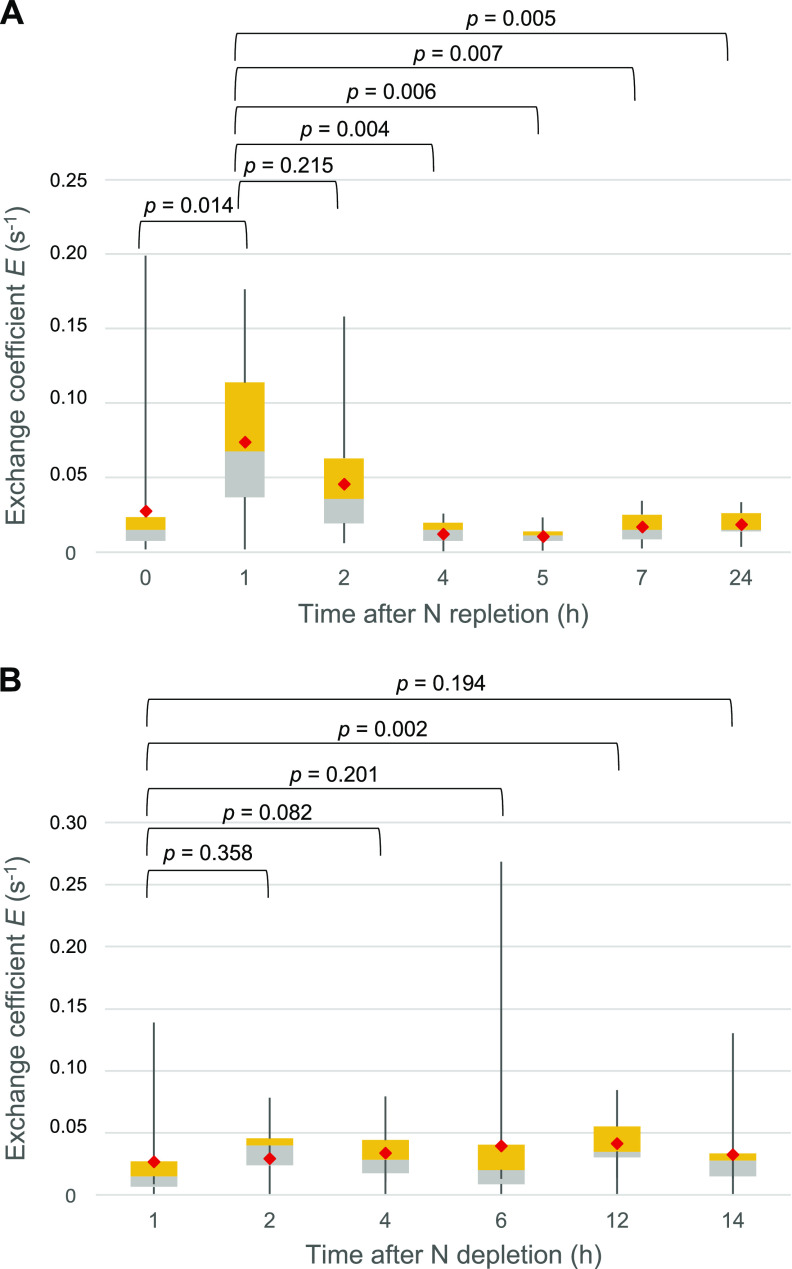
Calcein FRAP analysis after N replenishment and depletion. The exchange coefficient *E* (s^−1^) between vegetative cells was measured using confocal FRAP and values were plotted as box plot and statistically analyzed. (A) Filaments were grown in BG11_0_ medium for 72 h, washed, transferred into BG11 medium, and used for calcein labeling and FRAP analysis as described in Materials and Methods. (B) Filaments grown in BG11 medium were washed three times with BG11_0_ medium, resuspended in BG11_0_ medium, and used for calcein labeling and FRAP analysis. The difference between time points was assessed by the Mann-Whitney U test (*P* values shown at the top). Gray, quartile group 2 (from Q1 to median); yellow, quartile group 3 (from median to Q3); red diamonds, mean values.

Results presented in the previous sections were obtained with filaments that had been grown in BG11 medium or incubated for 48 h in BG11_0_ medium. In an independent approach, we addressed intercellular molecular exchange between vegetative cells after replenishing nitrate to N-depleted filaments or during acclimation to N deficiency, and in this case, exchange coefficients (*E*; s^−1^) were determined. A relatively low exchange coefficient was observed in cells that had been grown for 72 h in BG11_0_ medium, which increased after 1 h of incubation in BG11 medium (Fig. 4A). The exchange coefficient decreased thereafter, becoming very low 4 h after transfer to BG11 medium, a progression consistent with the time needed for the nitrate assimilation system to develop (24–26). Hence, the increase in *E* value observed after 1 h of incubation in BG11 medium appears to be in response to the change of medium rather than to nitrate assimilation, which indeed depresses intercellular molecular exchange. The reason for the positive effect of transfer to BG11 medium (BG11_0_ medium + 17.6 mM NaNO_3_), e.g., some kind of N signaling or salt-related effect, is unknown. On the other hand, when filaments grown in BG11 medium were transferred to BG11_0_ medium, a relatively rapid (1 to 2 h) increase in exchange coefficient was observed, but its statistical significance was obscured by the dispersal of the data (Fig. 4B).

**FIG 5:**
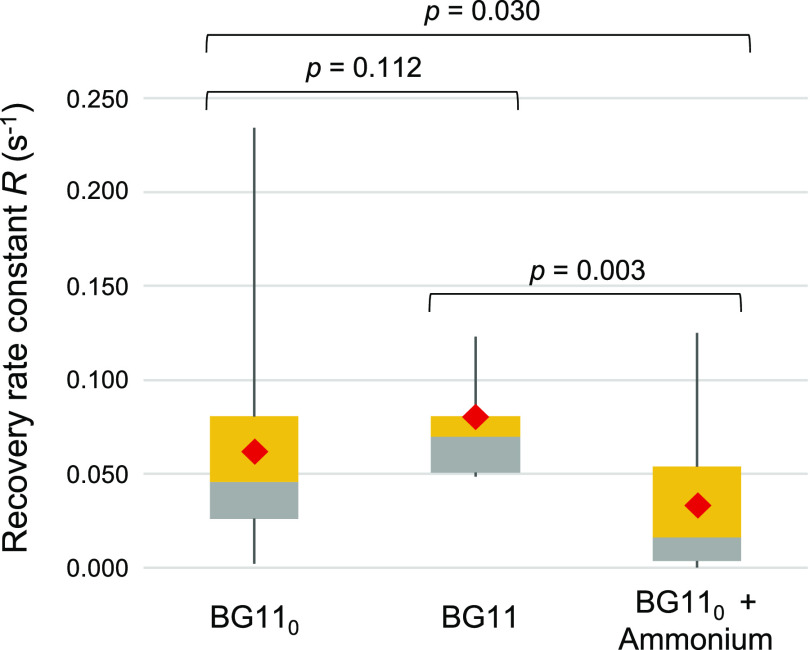
Calcein FRAP analysis after transfer of diazotrophically grown *Anabaena* to nitrate or ammonium-containing solid medium. Filaments grown in BG11_0_ medium were labeled with calcein in BG11_0_ medium and spotted on plates containing BG11_0_, BG11, or BG11_0_ plus NH_4_^+^ medium to carry out FRAP analysis. The recovery constant *R* (s^−1^) between vegetative cells was measured, represented in a box plot and subjected to the Mann-Whitney U test (*P* values shown at the top) to assess differences between conditions. Gray, quartile group 2 (from Q1 to median); yellow, quartile group 3 (from median to Q3); red diamonds, mean values. The numbers of cells subjected to FRAP analysis were 22 in BG11_0_, 9 in BG11, and 16 in BG11_0_ plus NH_4_^+^. Numbers of noncommunicating cells (*R* ≤ 0.01 s^−1^) were two in BG11_0_ medium and six in BG11_0_ plus NH_4_^+^ medium.

In order to explore possible quicker responses to combined N replenishment, we spotted calcein-labeled BG11_0_-grown filaments on plates with solid BG11, BG11_0_ plus NH_4_^+^, or (as a control) BG11_0_ medium and performed FRAP analysis without further incubation under culture conditions (Fig. 5). (Note that in all experiments described above, FRAP analysis was performed on cells spotted in solid medium of the same composition as the indicated growth or incubation medium [see Materials and Methods for further details].) Diazotrophically grown filaments spotted on BG11 medium showed somewhat increased recovery rates compared to those of filaments spotted onto BG11_0_ medium, which is reminiscent of the positive effect described above for the exchange coefficient at 1-h incubation in the nitrate-replenishing experiment (Fig. 4A). Because no time for induction of the nitrate assimilation system was allowed in this new experiment, this observation corroborates that there is a positive effect of changing from BG11_0_ to BG11 medium that is not related to nitrate assimilation. In contrast, when the diazotrophically grown cells were spotted onto BG11_0_ plus NH_4_^+^ medium, a clear decrease in the recovery rate constant took place (Fig. 5). Interestingly, a substantial number of noncommunicating cells (37.5%) was observed in this experiment only in the ammonium-containing plates. Because BG11_0_-grown cells are fully active in the assimilation of ammonium (i.e., they express at high levels the genes encoding glutamine synthetase and the ammonium transporters [27, 28]), these results likely reflect an effect of ammonium assimilation on the activity of septal junctions.

## DISCUSSION

Intercellular molecular exchange in the filaments of *Anabaena* takes place by diffusion through septal junctions, which are proteinaceous complexes that join the adjacent cells in the filament. The nanopores are likely the structures through which septal junctions traverse the septal PG (14, 29). Mutants lacking septal proteins SepJ and/or FraCD are impaired in intercellular molecular exchange and show a decreased number of nanopores (8). Here, we have shown that in cultures incubated in the absence of combined nitrogen (in which few noncommunicating cells were detected in the wild type), the rate of intercellular calcein transfer shows a strong correlation with the number of nanopores per septal PG disk. This supports the idea that the nanopores hold the septal junctions and suggests that the number of septal junctions corresponds to the number of nanopores.

The number of nanopores per septal PG disk in *Anabaena* sp. strain PCC 7120 is approximately 40 to 60 (Fig. 1C) (see also references 8, 17, and 20), but it has been reported to be approximately 155 in Nostoc punctiforme ATCC 29133 (13) or from 100 to 250 (described as microplasmodesmata) in the septa between vegetative cells of different heterocystous cyanobacteria, including Anabaena cylindrica Lemm., Anabaena variabilis (IUCC B377), *A. variabilis* Kütz (ATCC 29413), and Nostoc muscorum (30). In this and previous works (14, 16), it has been found that under certain conditions, *Anabaena* filaments contain a fraction of noncommunicating cells. It should be understood that a noncommunicating cell will have all (or nearly all) of its septal junctions closed in the septa at both cell poles. This suggests the existence of regulatory mechanisms that determine the closing or opening of all (or most) of the junctions in a cell depending on physiological conditions. Cryo-electron tomographic structures of *Anabaena* septal junctions *in situ* suggest a gating mechanism for reversible closure of the channel by a conformational change in a cytoplasmic cap (16). These junctions involve FraCD, and the channels (intercellular tubes) remaining in the Δ*fraC* Δ*fraD* mutant are reported to have lost regulation (16). We have observed that the Δ*fraC* Δ*fraD* mutant (CSVT22) still has noncommunicating cells, as is also the case for the Δ*sepJ* mutant (CSVM34) and the triple Δ*sepJ* Δ*fraC* Δ*fraD* mutant (CSVM141) (see Fig. S2 in the supplemental material). Whereas the noncommunicating cells in the Δ*fraC* Δ*fraD* and the triple Δ*sepJ* Δ*fraC* Δ*fraD* mutants may correspond to cells that have a very low number of nanopores (Fig. S1), the noncommunicating cells in the Δ*sepJ* mutant may correspond to cells in which the FraCD-septal junctions are closed. It should be noted that the Δ*sepJ* mutant contains approximately 40% of the number of nanopores (and, hence, of septal junctions) as observed in the wild type (Fig. 1), but these junctions can still be subject to gating (16).

Noncommunicating cells have been induced by different stresses, including treatment with a protonophore (16), or have been observed as aging heterocysts (8) or in mutants of proteins (specifically, sucrose transporters) that may affect the regulation of the septal junctions (14). Here, however, we have shown that noncommunicating cells can coexist with communicating cells in normal cultures and even within single filaments. The fact that communicating cells are more abundant in the absence than in the presence of combined nitrogen is consistent with the need of intercellular molecular exchange for diazotrophic growth and suggests a regulation of the activity of septal junctions in the context of the development of a diazotrophic filament. Indeed, we have previously shown that conditions that lead to induction of heterocysts and diazotrophic growth result in a substantial increase of the exchange coefficient (11). Reciprocally, here we have shown that conditions of nitrate or ammonium assimilation rapidly result in a decrease of the exchange coefficient or of the recovery rate constant (Fig. 4A and 5). Hence, septal junction activity appears to be regulated by the N status of the cells. The genes encoding the septal proteins SepJ, FraC, and FraD are constitutive or induced under nitrogen deprivation only at a low level. Thus, *sepJ* is induced approximately 1.5-fold to 2-fold under nitrogen deprivation (31–33), whereas *fraC* and *fraD* have been reported to be expressed constitutively (15, 33) or induced approximately 2-fold under nitrogen deprivation (32). These observations, together with the rapid response to combined N described here, are consistent with a rapid regulation of the septal junctions by a mechanism of gating, as previously suggested (16), rather than with strong induction/repression mechanisms.

The presence of individual noncommunicating cells in vegetative filaments suggests that the activity of septal junctions is a cell-level decision. We suggest that the metabolic status of an individual cell (e.g., its carbon-to-nitrogen balance) influences the activity of its septal junctions. In this model, cells with well-balanced metabolism keep their septal junctions closed, while cells that are experiencing metabolic imbalance open their septal junctions in order to pool metabolites with their neighbors. In cultures supplied with combined nitrogen, a link between metabolic status and septal junction activity leads to a stochastic incidence of communicating and noncommunicating cells as observed (Fig. 2). When combined nitrogen is withdrawn from the medium, all cells in the filament experience metabolic stress, leading to a general opening of septal junctions and faster molecular exchange, as previously observed (11). During diazotrophic growth, intercellular communication will remain rapid and the incidence of noncommunicating cells will remain low, because all vegetative cells are dependent on a supply of combined nitrogen from the heterocysts: any cell that starts to close its septal junctions will begin to experience metabolic stress and will therefore reopen them. The situation changes when ammonium is restored to the medium, because cells can then close their septal junctions without interrupting their nitrogen supply. This can account for the decrease in intercellular communication that we have observed in this situation (Fig. 5). When nitrate is restored to the medium, the delay in this switch (Fig. 4A) is consistent with the time required to establish the nitrate assimilation system in the vegetative cells. We note that the closure of septal junctions in vegetative cells following the restoration of combined nitrogen to the medium would deprive the heterocysts of their supply of carbohydrate and therefore would be expected to lead to rapid elimination of heterocysts from the filaments.

## MATERIALS AND METHODS

### Strains and culture conditions.

*Anabaena* (also known as *Nostoc*) sp. strain PCC 7120 was grown photoautotrophically in liquid BG11 medium (containing NaNO_3_ as the nitrogen source and in which ferric ammonium citrate of the original recipe [34] is replaced with ferric citrate), BG11_0_ medium (lacking NaNO_3_), or BG11_0_ plus NH_4_^+^ medium (containing 4 mM NH_4_Cl and 8 mM *N*-tris(hydroxymethyl)methyl-2-aminoethanesulfonic acid [TES]-NaOH buffer, pH 7.5) under constant white light (15 to 25 μE·m^−2^ s^−1^) with continuous shaking at 30°C. To induce heterocyst formation, filaments of an exponentially growing culture were harvested by centrifugation, washed three times in BG11_0_ medium, and resuspended in this medium. Plates of solid medium were prepared with 1% separately autoclaved agar and BG11, BG11_0_, or BG11_0_ plus NH_4_^+^ medium. *Anabaena* strains CSVM34 (Δ*sepJ* [18]), CSVT22 (Δ*fraC* Δ*fraD* [19]), and CSVM141 (Δ*sepJ* Δ*fraC* Δ*fraD* [8]) are markerless deletion mutants of strain PCC 7120, and they were routinely grown in BG11 medium.

### Peptidoglycan sacculi isolation and visualization.

Filaments grown in BG11 or BG11_0_ medium to approximately 3 to 4 μg chlorophyll *a*·ml^−1^ were harvested by centrifugation, and the sacculi were isolated by protease treatment and hot detergent extraction as described previously (8, 13). The purified sacculi were deposited on Formvar-carbon film-coated copper grids, and stained with 1% (wt/vol) uranyl acetate. All the samples were examined with a Zeiss LIBRA 120 PLUS electron microscope at 120 kV.

### FRAP analysis.

The aliquots (0.5 ml) taken from the cultures were incubated for 90 min with 10 μl calcein acetoxymethyl (AM) (1 mg/ml in dimethyl sulfoxide; Molecular Probes, Invitrogen) under constant shaking in the dark at 30°C. Filaments were then harvested by gentle centrifugation to prevent fragmentation, washed with BG11, BG11_0_, or BG11_0_ plus NH_4_^+^ medium as appropriate, resuspended in 1 ml fresh BG11, BG11_0_, or BG11_0_ plus NH_4_^+^ medium without the dye, and incubated for an additional 60 min under the same conditions. After the second incubation step, an aliquot was spotted onto a dry BG11, BG11_0_, or BG11_0_ plus NH_4_^+^ medium agar plate, and filaments were allowed to settle down by drying off excess liquid.

Small agar blocks with labeled filaments were transferred to a custom-built and temperature-controlled sample holder. During the FRAP experiments, the temperature was kept at 30°C. In the experiments described in Fig. 2, 3, and 5, cells were imaged with a Leica HCX PL Apo 63×, 1.4 numerical aperture (NA) oil immersion lens objective attached to a Leica TCS SP2 confocal laser scanning microscope with a 488-nm argon laser as the excitation source; fluorescent emission was monitored by collection across windows of 500 to 520 nm and through a 150-μm pinhole. In the experiments described in Fig. 4, a Nikon PCM2000 confocal laser scanning microscope equipped with a 100-mW argon laser was used. Samples were imaged using a 60× oil immersion lens objective, and the dye and pigments of the thylakoid membrane were simultaneously excited at 488 nm. The emission was separated into autofluorescence by a Schott RG665 red-glass filter and calcein fluorescence by an interference band-pass filter allowing transmission between 500 to 527 nm, and a pinhole of 50 μm was selected. After acquiring one or two prebleach images, the bleaching was carried out by an automated FRAP routine as previously reported (11). Postbleach images were taken in XY-mode approximately every 2 s over a time of 20 to 30 s (Fig. 2, 3, and 5) or every 3 s over a time period of 30 s (Fig. 4). For FRAP data analysis, kinetics of transfer of the fluorescent tracer was quantified and the exchange coefficient (*E*) or recovery rate constant (*R*) was calculated as previously described (11, 20).

### Data availability.

Original septal disk micrographs and FRAP raw data will be made available upon request.

10.1128/mSphere.01091-20.1 FIG S1 Frequency distributions of nanopore numbers and diameters in septal PG disks of wild-type *Anabaena* and Δ*sepJ* (strain CSVM34), Δ*fraC* Δ*fraD* (CSVT22), and Δ*sepJ* Δ*fraC* Δ*fraD* (CSVM141) mutants grown in BG11 medium. (A) Numbers of nanopores are organized in groups of 5 (from 0 to 5, from 5.01 to 10, etc.). (B) Nanopore diameters are organized in groups of 2 nm (from 0 to 2, from 2.01 to 4, etc.). Download FIG S1, PDF file, 0.01 MB. Copyright © 2021 Arévalo et al. 2021 Arévalo et al. This content is distributed under the terms of the Creative Commons Attribution 4.0 International license.

10.1128/mSphere.01091-20.2 FIG S2 Intercellular transfer of calcein between vegetative cells of *Anabaena* mutant strains CSVT22 (Δ*fraC* Δ*fraD*), CSVM34 (Δ*sepJ*), and CSVM141 (Δ*fraC* Δ*fraD* Δ*sepJ*) grown in BG11 medium (blue bars) or incubated for 48 h in BG11_0_ medium (orange bars). Cells analyzed by FRAP are organized in groups according to *R* values in increments of 0.01 s^−1^ (i.e., ≤0.01, 0.011 to 0.02, 0.021 to 0.03, …, >0.2 s^−1^). Data for wild-type *Anabaena* are presented in Fig. 2. Download FIG S2, PDF file, 0.01 MB. Copyright © 2021 Arévalo et al. 2021 Arévalo et al. This content is distributed under the terms of the Creative Commons Attribution 4.0 International license.
