# Supplementary figures and images for: Coexistence of Communicating and Noncommunicating Cells in the Filamentous Cyanobacterium Anabaena
Source: mSphere. 2021 Jan 13;6(1):e01091-20. doi: 10.1128/mSphere.01091-20 (PMC7845620; doi:10.1128/mSphere.01091-20)

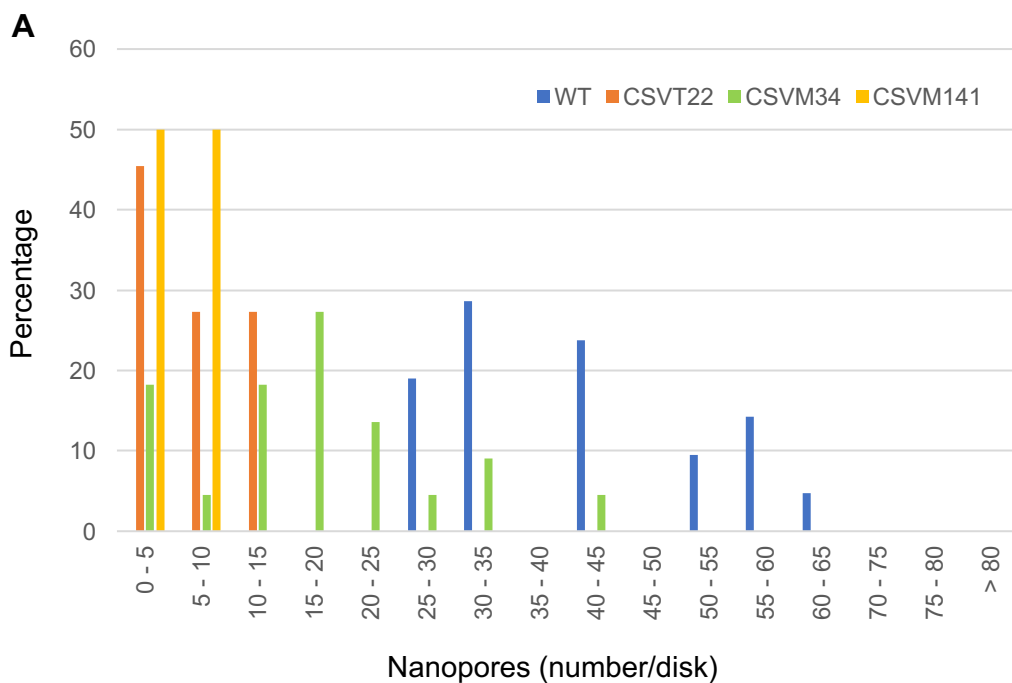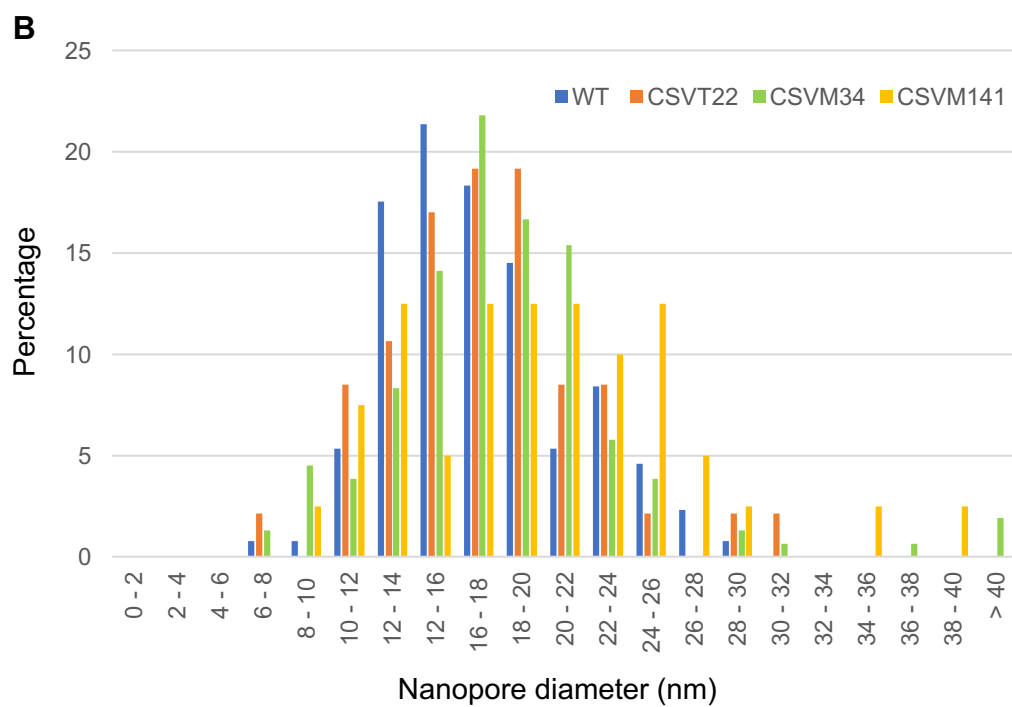

Supplement: FIG S1 [file mSphere.01091-20_sf001.pdf]

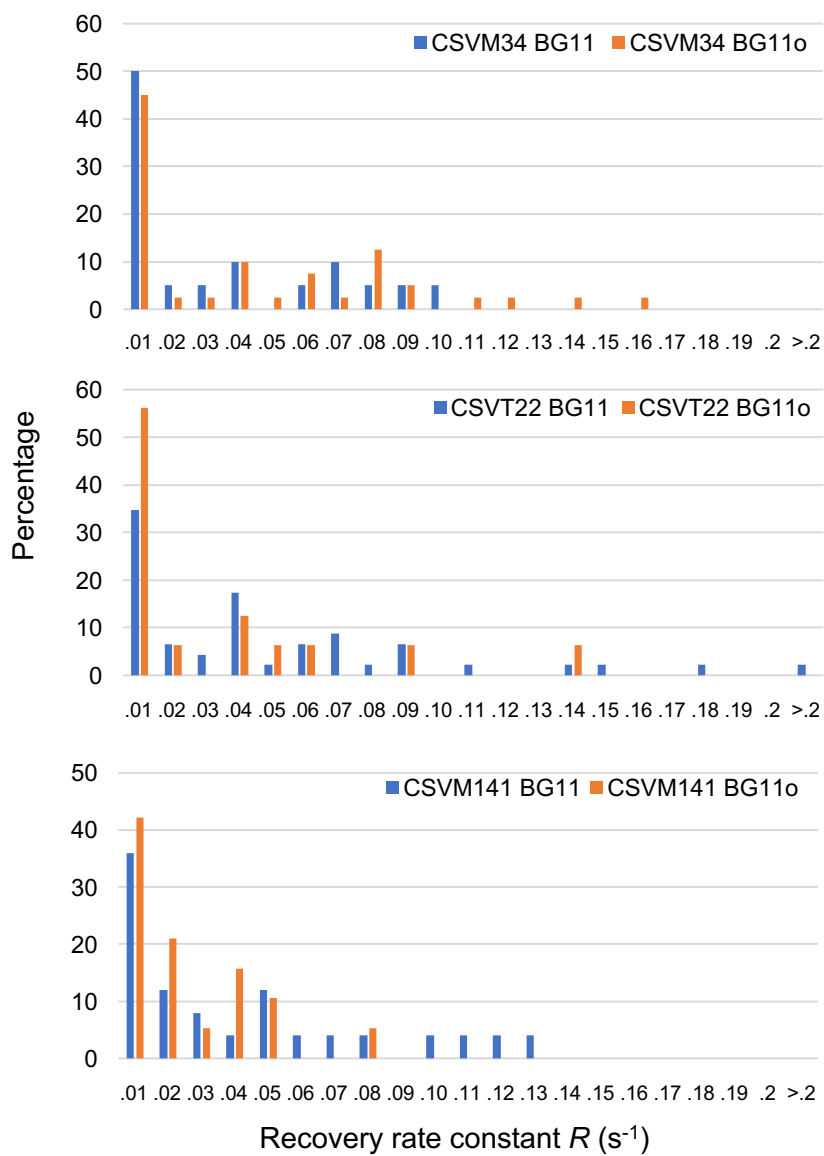

Supplement: FIG S2 [file mSphere.01091-20_sf002.pdf]
